# Sustainability of livestock farms: The case of the district of Moyobamba, Peru

**DOI:** 10.1016/j.heliyon.2023.e13153

**Published:** 2023-01-21

**Authors:** Giovanna Patricia Torres Jara de García, Luz Marlene Durand-Chávez, Hurley Abel Quispe-Ccasa, Jaime Lizardo Linares-Rivera, Gleni Tatiana Segura Portocarrero, René Calderón Tito, Héctor Vladimir Vásquez Pérez, Jorge Luis Maicelo Quintana, Gustavo Ampuero-Trigoso, Rafael René Robles Rodríguez, José Américo Saucedo-Uriarte

**Affiliations:** a Universidad Católica Sede Sapientiae, Nueva Cajamarca, San Martín, 22845, Peru; b Estación Experimental Agraria El Porvenir del Instituto Nacional de Innovación Agraria – INIA, Juan Guerra, San Martín, 22400, Peru; c Instituto Qualitas - Gestión, Innovación y Mejora Continua, Tarapoto, San Martín, 22201, Peru; d Facultad de Ingeniería Zootecnista, Agronegocios y Biotecnología de la Universidad Nacional Toribio Rodríguez de Mendoza de Amazonas, Chachapoyas, Amazonas, 01001, Peru; e Facultad de Zootecnia de la Universidad Nacional Agraria de la Selva, Tingo María, Huánuco, 10131, Peru

**Keywords:** Amazon region, Conglomerates, Livestock, Soil quality, Sustainable development

## Abstract

The Peruvian Amazon is a geographical area with great biodiversity, where the main economic activities are agricultural crops and grazing animals. The evaluation of sustainability in production systems is based on the analysis of economic, environmental and social components, which are variable between production units or livestock farms. The classification of livestock farms based on their characteristics of similarity and differences can contribute to the most appropriate assessment of their level of sustainability. The objective of this research was to determine the level of sustainability of livestock farms in the district of Moyobamba, San Martín, Peru, based on environmental, economic and social criteria. The research was carried out from November 2018 to February 2019 with a survey of a sample of 60 livestock farms out of a population of 2220. A survey-type form and data collection in the field were applied, adapting a methodology that proposes inferring on 33 indicators grouped into six criteria: three environmental criteria (soil quality, pasture health and animal quality), two economic criteria (farm system and farm economy), and a social criterion of the farm. A scale from 1 to 10 was used to assess the condition of each indicator. The typification of farms was carried out through a Conglomerate Analysis. To analyze the level of sustainability, Amoeba graphs were constructed for each defined farm group. Qualitative variables were analyzed with contingency tables and quantitative variables using the T test (p < 0.05). Three types of livestock farms were identified, differentiated by level of education, farm size, years in cattle raising and number of cattle heads (p < 0.05), where Group 1 is less experienced, Group 1 has more area and cattle, and Group 3 only have older years in livestock. There were significant differences between the evaluated criteria and the sustainability index. From the typification of livestock farms, Group 2 (13 farms) presented a higher level of sustainability as did Group 3 (16 farms), while Group 1 (31 farms) presented unsustainable conditions. The environmental indicators based on animal quality and farm system show unsustainability in all farms the District of Moyobamba, as they fail to exceed the threshold of sustainability (5).

## 1 Introduction

Sustainable development is a process of human societies seeking to meet the current needs of their generations, without compromising the capabilities of future generations to meet them. It seeks to improve the economic and social aspect of these interested societies while maintaining future capacities through the conservation of their natural resources [1,2]. The sustainability of production systems can be covered from the environmental, social and economic approach. Environmental sustainability seeks to develop human activities in harmony with the conservation of the environment, ecosystems and biodiversity [3]. A rational use of natural resources is proposed and to avoid the depletion of non-renewable resources, as well as, to avoid the generation of waste and the emission of pollutants [4]. From the social approach, it proposes to preserve the social and cultural network, as well as the ability to maintain common interests through democratic and non-exclusive means [5]. In the process of change towards a fairer society, education, health and peace are valued. The economic approach basically seeks to develop the financial capacity and profitability of human activities, conserving the natural resource base [6].

Sustainability at livestock farms is based on meeting the three basic aspects reviewed in sustainable development. In the economic aspect, it is sought that the productive activity is profitable and can generate profits for the farmers for the development of their productive systems and in parallel of their quality of life. In the environmental aspect, the reduction of air and water pollution is sought, avoiding the erosion of soil and pastures, and the conservation of productive quality of livestock, forests and biodiversity. In the social aspect, it seeks to improve access to social services and organizational capacity.

Livestock is one of the main economic activities in the Peruvian Amazon; therefore, it is the source of direct and indirect support for a large part of the national population. The Moyobamba district, in the province of Moyobamba, department of San Martín, has the largest population of livestock (16,476 heads), according to the 2012 National Agricultural Census [7]. The productive orientation of this segment is dual purpose (milk and meat), although the orientation for milk production is increasing recently, due to the growing demand for dairy products. However, the low efficiency of the productive indicators of these livestock systems is associated with problems such as: loss of competitiveness of producers, decreased productivity of pastures and forages, low carrying capacity of paddocks, low reproductive rates, among others [8]. Livestock farms in the Moyobamba district have adopted different technological levels to optimize their production processes, depending on their purchasing power. At the district level, in the 2220 livestock farms [7], the productive indicators are low, but the degradation of natural resources, population increase and growth of rural poverty have accelerated, which can give us signs of a crisis of unsustainability socio-environmental. Deforestation, fires, overexploitation of resources and non-conservationist agricultural practices can contribute to the intensification of this crisis, through the degradation of hydrographic basins and social-environmental conflicts [9].

The evaluation of sustainability can contribute to proposing strategies and making decisions regarding the productive processes developed in each cattle farm according to its characteristics. According to Altieri and Nicholls [10] and Altieri [11] it is possible to evaluate the sustainability of an agroecological system using a practical and simple methodology with indicators that can determine the state of the productive system. By evaluating each indicator, sustainability can be estimated in “Ameba” type diagrams the closer they get to the diameter of the circle (the optimum is a value of 10). The future of livestock farming lies in the level of sustainability in which agricultural farms are handling. Although the sustainability evaluation is based on the analysis of economic, environmental and social aspects, it is necessary to classify the farms according to their characteristics. The classification of livestock farms based on their characteristics of similarity and differences can contribute to the most appropriate assessment of their level of sustainability. Therefore, the research aimed to typify the farms of the district of Moyobamba, San Martín to assess their level of sustainability.

## 2 Material and methods

### 2.1 Studio location

**Fig. 1:**
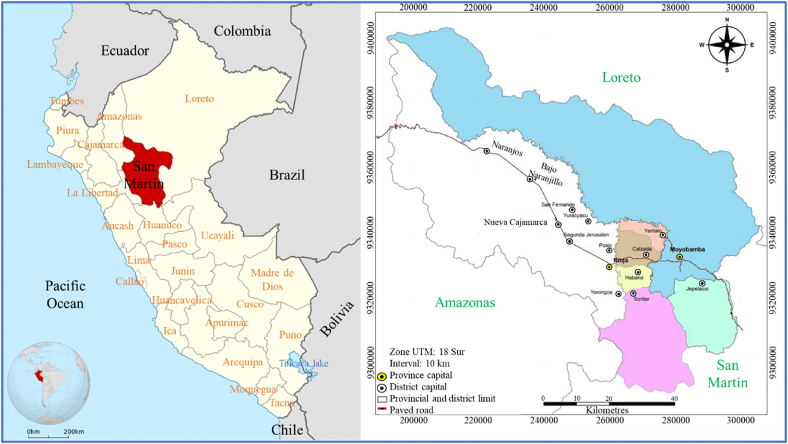
Research sampling location map.

The research was carried out from November 2018 to February 2019, in the district of Moyobamba, province of Moyobamba, department of San Martín (Fig. 1). The district has an extension of 2,737.57 km^2^, and an estimated population of 92,000 inhabitants. It is located between 5°26′33″ to 6°25′50″ south latitude and between 77°37′50″ to 76°41′25″ west longitude, at an altitude of 860 m above sea level. It presents a maximum annual average temperature of 28.4 °C and a minimum of 16.4 °C, average annual rainfall of 1487 mm and relative humidity of 84% (SENAMHI, 2017). The area is classified as Pre Montane-Tropical Humid Forest.

The trees with the greatest predominance in the livestock farms of the Moyobaba district are composed of *Ocotea quixos* (Ishpingo), *Cedrelinga catenaeformis* (Tornillo) and *Colubrina glandulosa* (Shaina), *Eucalyptus* sp (Eucalipto), *Ormosia coccinea* (Guayruro) and the most frequent fruit trees are *Inga edulis* (Guaba), *Psidium* sp (Guayaba) y *Mangifera indica* (Mango). The grass forage species with the greatest predominance are *Digitaria Decumbens* (Pangola), *Brachiaria brizantha* (Brizantha), *Brachiaria decumbens* (Decumbens), *Axonopus compressus* (Torourco) and *Paspalum dilatatum* (Pasto de agua) and legumes are *Arachis pintoi* (Maní forrajero) and *Pueraria phaseoloides* (Kutzú) [12].

In the livestock farms, breeds of Bos indicus cattle were identified as Brahman and Gyr, Bos taurus cattle, there are Brown Swiss and Simmental, and their crosses [13].

### 2.2 Sample and sampling

The sample was estimated based on the total population of livestock farms in the Moyobamba district, which were 2220 for the year 2012, with a population of 16,476 heads of livestock [7]. The sample size was calculated using the sample size formula for finite populations [14], using a confidence level of 95%, probability of success 80%, error 20% and estimation error 10%. A sample of 60 livestock farms was obtained and the sampling was by quotas, considering the farmers dedicated to livestock as the inclusion criteria and the availability of the farmers to be interviewed as the selection quota.

$$n=\frac{N*z_{\propto }^{2}*p*q}{e^{2}*\left(N-1\right)+z_{\propto }^{2}*p*q}$$

where, n = sample size, N = population size, Z = confidence level of 95%, e = estimation error 10%, p = probability of success 80%, q = probability of error 20% (1-p).

### 2.3 Data collection and indicators

A survey-type form and data collection in the field were applied, adapting a methodology that proposes inferring about soil quality, pasture health, animal quality, farm system, economic aspect and social aspect, through indicators. The validation of indicators was carried out adapting the methodology based on Altieri [11], Altieri and Nicholls [10], and Nicholls et al. [15]. The methodology proposed by Araujo et al. [16], which is based on the perception of the indicators that show us a level of its state. This methodology consisted of assigning qualitative values according to their condition. In a condition between least desired to moderate, it could receive values between 1 and 5; between moderate and desired, it could receive values between 5 and 10; that is, 1 represents the worst or least desired condition, while 10 represents the ideal or most desired condition.

Thirty-three indicators were evaluated, which were classified into six criteria: three environmental criteria (soil quality, pasture health and animal quality), two economic criteria (farm system and farm economy), and a social criterion of the farm. The description of each indicator can be seen in Table 1:

**Table 1:** Indicators of the environmental, economic and social criteria, for the evaluation of sustainability in livestock farms in the district of Moyobamba, San Martín.

Dimension	Criterion	Indicator	Characteristic
Environmental	Soil quality	Compaction	Compacted soil (1), moderately compacted (5), not compacted (10).
		Depth of the arable layer of the soil	Exposed soil with rocky views (1), thin layer of soil (5), Top soil >50 cm (10).
		Organic matter color	Pale, absence of OM (1), light brown, some presence of OM (5), dark brown, abundant OM (10).
		Soil cover	Without coverage, 100% exposed (1), soil with less than 50% covered (5), more than 50% covered (10).
		Erosion	Severe erosion (1), low levels of erosion (5), absence of erosion symptoms (10).
		Presence of invertebrates	Absence of invertebrate activities (1), few earthworms and arthropods present (2), abundant presence of invertebrates (10).
		Microbiological activity	Very little effervescence to hydrogen peroxide (1), slight effervescence (5), abundant effervescence (10).
	Pasture health	Appearance	Chloritic (1), Light green color with some loss of pigment (5), Dark green color, without deficiency symptoms (10).
		Pasture growth	Uneven rootstock, limited growth (1), denser, more uniform rootstock (5), vigorous growth (10).
		Weeds presence	More than 50% weeds (1), between 6 and 15% weeds (5), less than 6% weeds (10).
		Annual or potential return	Low in relation to the ideal mean (1), acceptable, in relation to the ideal mean (5), high, in relation to the ideal mean (10).
		Vegetation diversity	A single pasture species (1), 2–3 pasture species (5), > than 4 pasture species (10).
		Paddocks use	With a single paddock (1), with 2–3 paddocks (5), with more than 5 paddocks (10).
		Trees presence	No trees (1), < than 5 trees per paddock (5), > than 10 trees per paddock (10).
	Animal quality	Calving per year	< than 70% calving equal to the number of cows (1), 70–80% calving equal to the number of cows (5), 90–100% calving equal to the number of cows (10).
		Animal performance	State of malnutrition (1), moderate state (5), excellent state of the animal (10).
		Animal breeds	Undefined crossbreeds (1), F1 or defined crossbreeds (5), specialized breeds (10).
		Selling weight	< than 200 kilos of live weight (1), Between 200 and 300 kilos of live weight (5), > than 400 kilos of live weight (10).
Economic	Farm system	Water supply	Rain reservoirs (1), uses rivers or streams (5), has a water pipe system (10).
		Sanitary facilities	It does not have a latrine (1), It has a latrine, a cesspool (5), It has a drainage system, a cesspool (10).
		Forest areas	It does not have forest reserves (1), It has a forest reserve without wood species (5), It has a forest reserve with wood species and wild animals (10).
		Reforestation	No reforestation (1), Little reforestation (5), Regular reforestation in crops and free areas (10).
		Crop diversity	A single crop (1), Few annual and permanent crops (5), Diversified with annual and permanent crops (10)
	Farm economics	Housing quality	Very humble, material from the area (1), regular, some comfort, with transformed materials (5), comfortable, with finishing materials (10).
		Tools and equipment	Basic tools, machete, axe, lampa (1), few non-basic tools (5), uses many tools per activity (10)
		Vehicle	Does not have (1), has non-motorized (5), has motorized (10).
		Income levels in livestock	< than the minimum salary (930/month) (1), At the minimum (5), > the minimum salary (10).
		Handling infrastructure	It does not have (1), It only has a pen (5), It has a pen, and a handling sleeve (10).
		Other income	Does not have (1), temporarily performs tasks outside his farm (5), works simultaneously, has permanent external work (10).
		Hire staff	Does not hire (1), temporarily hires (5), permanently hires staff (10).
		Bank credit	He does not access credit (1), he has credit for personal expenses (5), he has bank credit for investment in his farm (10).
Social	Social aspects	Belongs to an organization	Does not belong (1), belongs, but does not participate (5), belongs and participates (10)
		Educational level of their sons	They do not study, or only primary (1), They study up to secondary school (5), They study university (10)

### 2.4 Farms classification and sustainability evaluation

Based on the 33 indicators, the farms were grouped according to their similarities, using a hierarchical cluster analysis. Using Ward’s method, to differentiate farmer with minimum variability within groups and maximum between groups, three types of farms were found. Then, the characteristics of the defined types of farms were described, highlighting the differentiating characteristics between groups. Finally, Ameba graphs were constructed to analyze the situation of each indicator and its influence on the sustainability of each group of defined farms. Based on the evaluation of the level of sustainability, proposals were made for each type of farm. Good livestock practices and agroecological handling were focused on [17].

### 2.5 Statistical analysis

The typification of farms was carried out through a Cluster Analysis, using the Ward method and Jaccard distance as a grouping technique. Variables were standardized for processing and grouping was performed using Jaccard’s distance by Ward’s method. The qualitative variables were analyzed with contingency tables and Chi-squared test, and the quantitative variables using the T-test (p < 0.05), in the Infostat v.2017 program. For the analysis of the dependent variables: farm size, hectares of pasture and level of sustainability by criterion, an ANOVA and the mean DGC test were used to determine the differences between farm types. The variables: farm size, pasture areas and years of livestock experience were continuous variables, but the number of heads of livestock were treated as continuous numerical variables, except the number of livestock from which they were transformed. ANOVA was performed to find differences between the groups with respect to the mentioned variables. These variables were significant (p < 0.05), which was applied a means test with Fisher’s statistic, to determine differences between the groups formed.

## 3 Results

To determine the existence of different types of farms, a cluster analysis was performed on 60 randomly selected farms. Six criteria and 33 indicators were considered: soil quality (7 indicators), pasture health (7 indicators), animal quality (4 indicators), farm system (5 indicators), economic (8 indicators), and social (2 indicators). Three groups of livestock farms were defined, where 51% were assigned to Group 1, 22% to Group 2 and 27% to Group 3 (Figure S1). According to Table 2, the level of education was associated with the type of farm (p < 0.05), where the primary level predominates in Group 1, the secondary level in Group 3, and the university level is similar in Groups 2 and 3. Significant differences were found between farm size and number of heads of livestock (p < 0.05), being higher in Group 2, with no differences between Groups 1 and 3. The years in livestock also varied between groups (p < 0.01), being higher in Group 2 and 3, compared to Group 1.

**Table 2:** Characteristics of the producer and the livestock farm, according to the type of farm in the district of Moyobamba, San Martin.

Indicator	p-value	Group 1	Group 2	Group 3
N		31	13	16
Degree of instruction**	0.02*			
Primary		80.65%	53.85%	37.50%
Secondary		19.35%	23.08%	37.50%
University		0.00%	23.08%	25.00%
Farm size (ha)	0.02*	14.10b	28.03a	14.03b
Pasture area (ha)	0.06	8.82	17.00	7.59
Years in livestock	<0.01**	16.02b	27.38a	27.63a
Number of livestock	0.02*	15.32b	24.15a	19.44b

Different letters in the same row indicate statistical differences at the p < 0.05 (*) or p < 0.01 (**) level according to T-test. The degree of instruction is a categorical variable analyzed with Chi-square (p < 0.05).

Significant differences were found between the means of the sustainability indicators, between the groups formed (Table 3). Regarding the classification criteria, the value results from the average of indicators that make up each criterion. The threshold of sustainability is based on a value of five (5), as it is considered as an intermediate point of an ideal condition. In Table 3, the quality of the soil in the farms of Group 2 has a higher average value with 8.77, resulting in a very favorable condition (near to 10). Similarly, pasture health, animal quality and general sustainability index are higher in this group. For the soil quality criterion, the three groups exceed the threshold of sustainability; however, several criteria have values less than five, and for the farm system, economic and social criteria, Group 1 had the lowest sustainability values. The general sustainability index is the average of the values of the classification criteria. Only Group 2 and Group 3 exceeded the sustainability threshold.

**Table 3:** Means (±SD) by classification criteria of sustainability indicators, according to type of farm in the district of Moyobamba, San Martin.

Criteria	Indicators	Group 1	Group 2	Group 3	p-value
Soil quality	7	7.27 ± 0.22b	8.77 ± 0.34a	6.42 ± 0.31b	<0.01
Pasture health	7	4.57 ± 0.13b	6.17 ± 0.20a	4.71 ± 0.18b	<0.01
Animal quality	4	2.59 ± 0.16b	4.27 ± 0.25a	2.56 ± 0.23b	<0.01
Farm system	5	3.15 ± 0.21b	4.97 ± 0.33a	4.78 ± 0.30a	<0.01
Farm economics	8	3.79 ± 0.23b	8.09 ± 0.35a	7.24 ± 0.32a	<0.01
Social aspects	2	3.26 ± 0.39b	5.27 ± 0.60a	4.88 ± 0.54a	<0.01
General sustainability index		4.11 ± 0.13c	6.26 ± 0.20a	5.10 ± 0.18b	<0.01

Different letters in the same row indicate statistical differences at the p < 0.01 level, according to Fisher’s test.

**Fig. 2:**
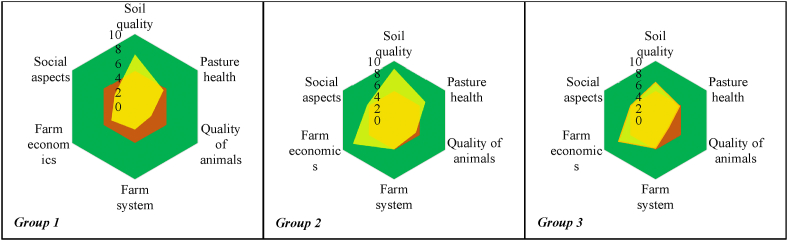
Ameba graphs for sustainability analysis according to six evaluation criteria, in three types of farms in the district of Moyobamba, San Martín.

Fig. 2 shows the sustainability level ameba graphs of the three types of livestock farms, based on the six evaluation criteria. The Group 1 farms show a graph that did not exceed the majority of the sustainability threshold (5) in all the criteria, with the exception of soil quality. In contrast, the graph of Group 2 farms covered most of the criteria threshold, with the exception of animal quality. In Group 3 farms, the graph is similar to Group 2, but with a lower index in pasture health, animal quality and farm system.

Based on Tables 3 and 4 presents the critical indicators in each group, according to sustainability evaluation criteria, which can guide the formulation of strategies for their proper handling. In soil quality, soil compaction fluctuated from 5.25 to 8.46 but, although compaction is higher in Group 3, the critical indicators are presence of invertebrates and microbiological activity. In pasture health, animal quality and Farm System, Table 3 shows that many indicators are limiting and affect the sustainability of farms. The diversity of vegetation is the indicator with the lowest indices (they fluctuate from 1.13 to 2.23), as well as the weight of bull sales (they fluctuate from 1.00 to 3.08).

**Table 4:** Critical indicators by evaluation criteria, according to type of farm in the district of Moyobamba, San Martin.

Criteria	Group 1 (31)	Group 2 (13)	Group 3 (16)
Soil quality			Presence of invertebrates and Microbiological activity
Pasture health	Pasture growth, Annual or potential return, Vegetation diversity and Presence of trees	Vegetation diversity and Presence of trees	Appearance, Pasture growth, Annual or potential return, Vegetation diversity and Use of paddocks
Animal quality	Calving per year, Animal performances, Animal breeds and Selling weight	Calving per year, Animal breeds and Selling weight	Calving per year, Animal performances, Animal breeds and Selling weight
Farm system	Water supply, Forest area, Reforestation and Crop diversity	Water supply and Reforestation	Sanitary facilities, Forest area, Reforestation and Crop diversity
Farm economics	Housing quality, Livestock income levels, Handling infrastructure, Hire staff and Bank credit		Housing quality and Handling infrastructure
Social aspects	Belongs to organization and Educational level of their sons	Belongs to organization	Belongs to organization

The economic indicators are not limiting in Group 2 farms, but in Groups 1 and 3, especially quality of housing and handling infrastructure. Belonging to an organization, in social indicators, is critical in all groups, becoming a weakness for sustainability. In Group 2, 8 critical indicators were found, but in Groups 3 and Group 1, there were 18 and 19 critical indicators, respectively.

## 4 Discussions

This is the first study that evaluates the level of sustainability in livestock farms in the Moyobamba district with a practical and simple evaluation methodology. The typification of farms through a multivariate cluster analysis is a useful strategy to determine groups of individuals with similar characteristics, within the group and more distant between groups [18,19,20]. The classification allows describing the main characteristics of the producers and farms in each defined group. The producers of Group 1, have mostly (>80%) primary education and <20% have secondary education. Maser et al. [21] argue that education is the basis for social sustainability; and that the weakness in school instruction could have a negative effect on the adoption of technological innovations, due to the difficulty of understanding ecological processes and suggested adaptive strategies, determining their level of sustainability [11,17]. The producers of groups 2 and 3 exceeded the values of the sustainability threshold, they have 25% of university education, which could facilitate the processes of technology adoption [22,23]. With respect to farm size and number of head of livestock, Group 2 farms show better conditions than the other groups. The farm size allows producers to have advantages over natural resources; furthermore, environmental sustainability can be based on the constant conservation of natural capital [11,24]. The typification of farms corresponds to the construction of groups based on the characteristics observed and that characterize them [25]. These main characteristics correspond to the level of sustainability. In Table 2, in the general sustainability index, only the Group 2 farms handling to exceed the sustainability threshold; while farms in Group 3 and 1 are still below the threshold. The evaluation of the sustainability of farms involves the analysis of the environmental, economic and social components together, as pillars that support the sustainability of the systems [26,27]. In this sense, the Group 1 farms would be unsustainable due to deficiencies in the economic, social and part of the environmental component. Under this perspective, the Group 3 farms comply with the economic component, but the social component is not sustainable; On the other hand, the Group 2 farms comply with most of the indicators, except for two criteria of the environmental component.

Environmental sustainability is based on the rational use of natural resources, on the absorption capacity of the ecosystem, on maintaining constant natural capital, preventive principles and laws that regulate the matter [11,28]. Regarding social sustainability, aspects such as: distributive equity, satisfaction of basic social services, gender equity, demographic stabilization and political participation are considered [21]. While economic sustainability includes sound macroeconomic management, economic growth with poverty reduction, strengthening of agri-food security, adaptation of the government’s role to eliminate distortions in the cost structure, where environmental and social costs must be included [29,30].

The environmental component was based on four criteria: soil quality, pasture health, animal quality, and farm system. For soil quality, all farms handling to exceed the sustainability threshold, except for the presence of invertebrates and microbiological activity, which are below the threshold in Group 3 farms. According to Araujo et al. [16] and, Altieri and Nicholls [31], soils are the most important component of agroecosystems, and their quality influences production. The health of the pastures is weak, since it shows unsustainability in some indicators. The farms in groups 1 and 3 do not handling to exceed the sustainability threshold (a value of 5), only the farms in group 2. The appearance, growth, presence of weeds and annual yield require handling strategies to improve these indicators. Acosta et al. [17] and Drewry et al. [32] suggest that pastures require recovery conditions, and adequate physical, chemical and biological conditions of the soil. The presence of trees improves plant diversity, even more so if soil fertility is limited. The strategy of incorporating trees allows the recovery of the soil from the reduction of compaction, improvement of aeration, water infiltration capacity and soil fertility [33,34], which are observable characteristics in silvopastoral systems. The quality of the animals is low in the farms of all groups, more noticeably in Groups 1 and 3. According to Sarabia et al. [35], despite adequate pasture handling, establishment of silvopastoral systems and handling during the dry season, without an efficient animal component, profitable livestock farming is a great challenge. The quality of the animals is a reflection of the condition of the rest of the components, such as pastures. Therefore, if the conditions of the pastures are improved, the productive and reproductive response of the animals could be improved [11,17,36,37]. Regarding the farm system indicators, none of the groups exceeded the sustainability threshold. The ability to have water on the farm is essential to increase the production of pastures and crops, therefore greater income for the producer [38]. The forest and reforestation indicators are related to the possibility of having sufficient areas for conservation and reforestation [23].

Within the economic component, the Group 2 farms exceeded the sustainability threshold, and have the highest indicators; however, Group 1 farms have serious limitations. The level of income depends on the functionality of the agroecosystems [11]. The economic sustainability of farms includes resource management and economic security in the short and medium term [29,30]. In the social component, the two indicators of difficulty in its sustainability are detailed (belonging to an organization and degree of education of the sons). The organization of producers is one of the main limitations for development, since the lack of an entity that represents and plans group improvement conditions leaves producers at the mercy of individual decisions, which will depend on their particular state [21]. This deficiency is related to the lack of leadership and credibility of the organizations [27].

The suggested proposals regarding the level of sustainability are based on the adoption of improvement techniques towards sustainable livestock. The environmental reconversion of livestock depends on the producers, and the biophysical characteristics of the farms [39]. Therefore, the transition towards the improvement of sustainability must be done with the participation of the producer and his capacities in infrastructure and availability of natural resources. The improvements are framed in the establishment of silvopastoral systems to counteract deficiencies in the quality of soils, pastures and animals, as an autonomous system in an integrative approach [40]; as well as the sustainable livestock approach towards a climate change scenario, which strengthens local governance with all the actors involved to achieve climate-smart livestock [17,34,41,42].

## 5 Conclusions

The livestock farms of the District of Moyobamba are classified into three different types and their level of sustainability varies among them. From the typification of livestock farms, Group 2 had a higher level of sustainability, while Group 1 had unsustainable conditions, and Group 3 had a medium level of sustainability. The environmental indicators based on animal quality and farm system show unsustainability in all farms, as they fail to exceed the threshold of sustainability (5). Strategies for improving soil conditions, pastures, and good livestock practices are decisive in contributing to the improvement of other economic and social indicators.

## Declarations

### Author contribution statement

Giovanna Patricia Torres Jara de García: Performed the survey; Conceived and designed the experiment; Contributed materials.

Luz Marlene Durand-Chávez: Performed the survey; Contributed materials; Analysis tools or data; Wrote the paper.

Hurley Abel Quispe-Ccasa: Conceived and designed the experiment; Analyzed and interpreted the data.

Jaime Lizardo Linares-Rivera: Conceived and designed the experiment; Performed the experiments; Analyzed and interpreted the data; Wrote the paper.

Gleni Tatiana Segura Portocarrero: Conceived and designed the experiment; Contributed materials; Wrote the paper.

René Calderón Tito: Conceived and designed the experiment; Analyzed and interpreted the data; Wrote the paper.

Héctor Vladimir Vásquez Pérez: Contributed materials and analysis tools or data; Analyzed and interpreted the data.

Jorge Luis Maicelo Quintana: Contributed materials; Analyzed and interpreted the data; Wrote the paper.

Gustavo Ampuero-Trigoso: Contributed materials; Analyzed and interpreted the data; Wrote the paper.

Rafael René Robles Rodríguez: Conceived and designed the experiment; Contributed materials; Wrote the paper.

José Américo Saucedo-Uriarte: Contributed materials; Analyzed and interpreted the data; Wrote the paper.

### Funding statement

This research did not receive any specific grant from funding agencies in the public, commercial, or not-for-profit sectors.

### Data availability statement

Data will be made available on request.

### Declaration of interests statement

The authors declare no conflict of interest.

### Additional information

The additional information is available for this paper.
