# Supplementary material for: Sustainability of livestock farms: The case of the district of Moyobamba, Peru
Source: Heliyon. 2023 Jan 21;9(2):e13153. doi: 10.1016/j.heliyon.2023.e13153 (PMC9900507; doi:10.1016/j.heliyon.2023.e13153)
Supplement: Multimedia component 1 [file mmc1.docx]

Supplementary information: **Sustainability of Livestock Farms: The case of the district of Moyobamba, Peru**


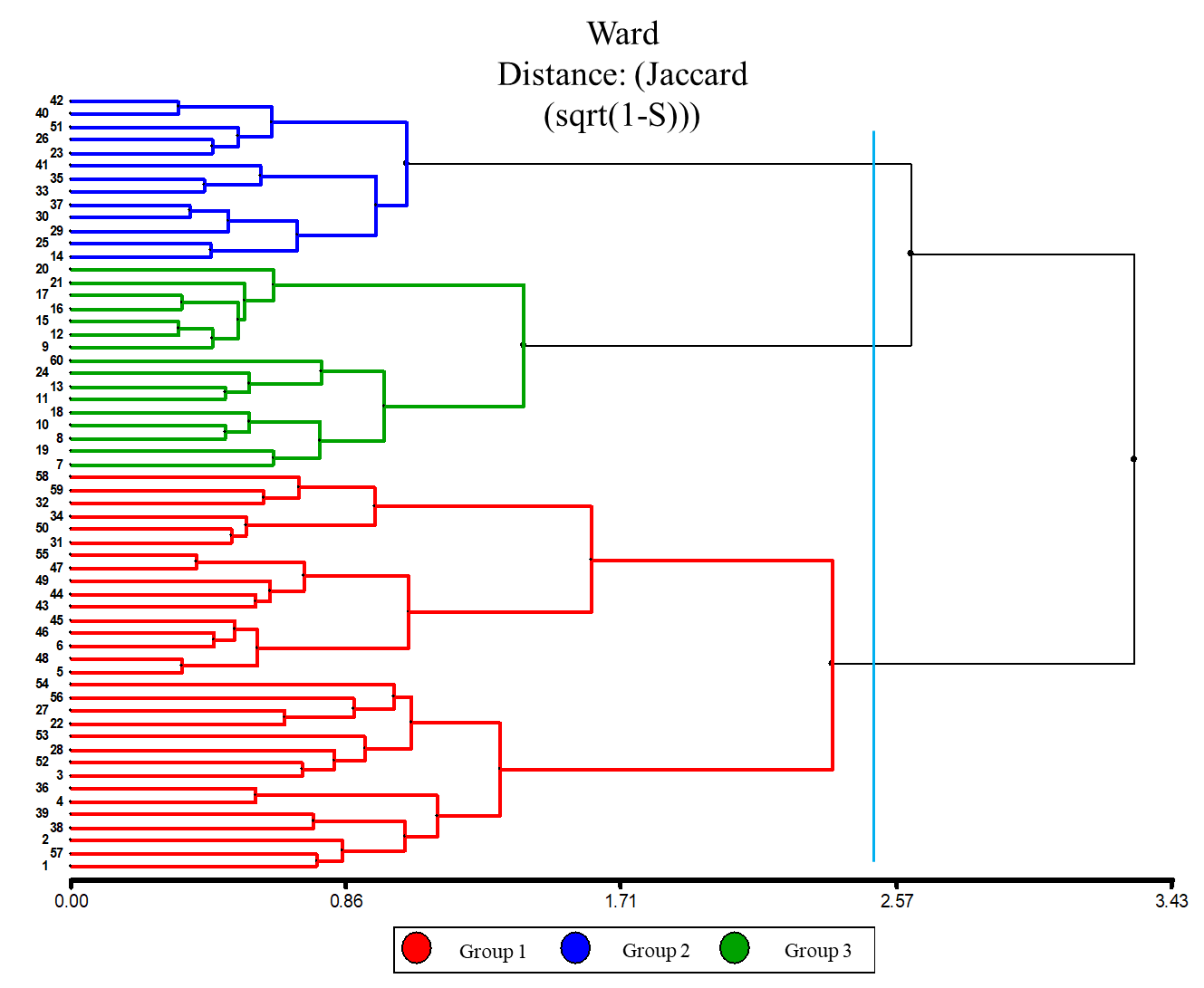


Figure 1S. Formation of farm groups according to Ward's method
